# Avian Cytochrome P450 (CYP) 1-3 Family Genes: Isoforms, Evolutionary Relationships, and mRNA Expression in Chicken Liver

**DOI:** 10.1371/journal.pone.0075689

**Published:** 2013-09-30

**Authors:** Kensuke P. Watanabe, Yusuke K. Kawai, Yoshinori Ikenaka, Minami Kawata, Shin-Ichi Ikushiro, Toshiyuki Sakaki, Mayumi Ishizuka

**Affiliations:** 1 Laboratory of Toxicology, School of Veterinary Medicine, Hokkaido University, Sapporo, Hokkaido, Japan; 2 Department of Biotechnology, Faculty of Engineering, Toyama Prefectural University, Toyama Prefecture, Japan; Weizmann Institute of Science, Israel

## Abstract

Cytochrome P450 (CYP) of chicken and other avian species have been studied primarily with microsomes or characterized by cloning and protein expression. However, the overall existing isoforms in avian *CYP1-3* families or dominant isoforms in avian xenobiotic metabolism have not yet been elucidated. In this study, we aimed to clarify and classify all of the existing isoforms of *CYP1-3* in avian species using available genome assemblies for chicken, zebra finch, and turkey. Furthermore, we performed qRT-PCR assay to identify dominant CYP genes in chicken liver. Our results suggested that avian xenobiotic-metabolizing *CYP* genes have undergone unique evolution such as *CYP2C* and *CYP3A* genes, which have undergone avian-specific gene duplications. qRT-PCR experiments showed that *CYP2C45* was the most highly expressed isoform in chicken liver, while *CYP2C23b* was the most highly induced gene by phenobarbital. Considering together with the result of further enzymatic characterization, CYP2C45 may have a dominant role in chicken xenobiotic metabolism due to the constitutive high expression levels, while CYP2C23a and CYP2C23b can be greatly induced by chicken xenobiotic receptor (CXR) activators. These findings will provide not only novel insights into avian xenobiotic metabolism, but also a basis for the further characterization of each *CYP* gene.

## Introduction and Aim

The cytochrome P450 (CYP) families 1–3 are the major xenobiotic-metabolizing enzymes that contribute to the bioactivation or inactivation of numerous xenobiotic compounds such as drugs and environmental chemicals [1]. Xenobiotic-metabolizing ability is an important factor in determining sensitivity to foreign chemical compounds because the metabolites are readily excreted into urine. Xenobiotic-metabolizing CYPs are known to be expressed primarily in liver. mRNA expression levels of CYPs are known to correlate with their protein levels and enzymatic activity, although the strength of the correlations depends on the CYP isoform [2], [3].

Approximately 10,000 avian species are currently known, including poultry species, such as chicken and quail, and wild bird species, such as water birds and raptors. Because these species have numerous opportunities for exposure to drugs, pesticides, and other environmental chemicals, their xenobiotic-metabolizing ability and its relationship with chemical sensitivity is an important field of research. We previously showed large differences in the CYP-mediated metabolic activity of warfarin in chicken, ostrich, mallard, and owl [4]. This study explained, in part, the inconsistency between high LD_50_ value in chicken and frequent reports of secondary poisoning incidents in wild bird species.

Studies of CYP-mediated xenobiotic-metabolizing abilities in avian species have primarily examined activities toward specific substrates using liver microsomes or *CYP* induction by specific agents [5], [6]. Recently, enzymatic characteristics and phylogenetic relationships within each family have been examined for the chicken CYP isoforms, i.e., CYP1, CYP2C, CYP2D49, and CYP3A37 [7], [8] [9], [10], [11]. However, the xenobiotic-metabolizing *CYP* genes in avian species have not been completely elucidated, and the importance of each isoform has not been evaluated in comparison with other isoforms. Knowledge of each isoform is needed to be integrated into the whole perspectives.

In this report, we aimed to elucidate the existing isoforms of the avian *CYP1-3* families using the available genome databases for chicken (*Gallus gallusdomesticus*), zebra finch ( *Taeniopygia* *guttata* ), and turkey ( *Meleagris* *gallopavo* ). We also investigated the phylogenetic relationships in comparison with human *CYPs*. Avian species are evolutionarily divided into three groups [12]. The Palaeognathae (e.g., ostrich and emu) diverged first, followed by the Galloanserae (e.g., chicken, turkey, and most other poultry species) and the Neoaves, which includes 99.5% of all avian species. Thus, comparative analyses of chicken, zebra finch, and turkey can provide a comprehensive understanding of the common features and differences among most bird species, except for those of the Palaeognathae. We used quantitative real-time RT-PCR (qRT-PCR) methods to clarify basal *CYP* mRNA expression patterns and phenobarbital (PB) induction in chicken liver to identify the dominant isoforms. Furthermore, we compared the enzymatic activity of chicken CYP2C proteins, i.e. CYP2C23a, CYP2C23b and CYP2C45. These results on avian xenobiotic-metabolizing CYP isoforms will serve as the basis for future studies of avian xenobiotic metabolism and species differences in the resulting chemical sensitivities.

## Materials and Methods

### Animals

Eight male and eight female White Leghorn chickens were obtained from Hokkaido Central Chicken Farm (Hokkaido, Japan). They were housed in plastic cages and fed a standard diet (Nihon Nosan Kogyo Co., Yokohama, Japan) and water *ad libitum*. The animal room was maintained at 25 °C ± 2 °C under a 12-h light–dark cycle (starting at 07:00 h). They were divided into two groups, i.e. saline-treated and PB-treated groups with each four individuals. For saline-treated group and PB-treated group, saline or PB (80 mg/kg dissolved in saline) were intraperitoneally injected for three days, respectively. The chickens were sacrificed after 24 hours of the last dose, at the age of 8 weeks old, using carbon dioxide, and their livers were collected. The livers were immediately frozen in liquid nitrogen and stored at −80 °C until use. All experiments using animals were performed under the supervision and with the approval of the Institutional Animal Care and Use Committee of Hokkaido University (Permit number 10-0067).

### Phylogeny and synteny analyses of *CYP1-3* genes from three avian species

The *CYP1-3* genes of chicken, zebra finch, and turkey were retrieved using an NCBI BLAST search. Because the first gene set contained *CYP* genes other than *CYP1-3*, we first analyzed the retrieved genes by neighbor-joining phylogeny to select the *CYP1-3* genes for further analyses. We then separated the genes into *CYP1-3* families and performed phylogenetic analysis. The deduced amino acid sequences were aligned using MUSCLE [13] and employed for model selection and construction of maximum likelihood trees (bootstrapping = 100) using MEGA5 [14]. Because preliminary nucleotide analysis provided very similar results for all *CYP1-3* families, we focused our study on the amino acid sequence results. Gaps and missing data were excluded from the analyses by partial deletion with site coverage cutoff of 95%.

NCBI’s MapViewer (http://www.ncbi.nlm.nih.gov/projects/mapview/) was used to visualize chromosomal synteny maps for each species. The latest genome assemblies were used; chicken Build 3.1, zebra finch Build 1.1, turkey Build 1.1, and human Build 37.3. UCSC BLAT (http://genome.ucsc.edu/index.html) and the Ensembl database (http://asia.ensembl.org/index.html) were used for additional confirmation of missing genes. Orthologous relationships were confirmed by synteny analysis. The gene sequences were submitted to the Cytochrome P450 Nomenclature Committee for nomenclature [15].

### Quantitative real-time RT-PCR

Total RNA was extracted from the chicken livers using RNeasy Mini Kits (Qiagen, Valencia, CA). The purity and quantity of RNA were determined spectrophotometrically using NanoDrop ND-1000 (Thermo Scientific, DE, USA). A260/280 and A260/230 were generally ≥2. Total RNA (2 µg) was reverse transcribed using ReverTra Ace (Toyobo, Tokyo, Japan) in a final volume of 40 µl, according to manufacturer’s instructions. Gene-specific qRT-PCR primers (Table S1) were synthesized by Sigma-Aldrich (Tokyo, Japan). qRT-PCR was performed using the StepOnePlus Real-Time PCR system (Applied Biosystems, Foster City, CA, USA). The 10-µl PCR reaction mixture consisted of Fast SYBR Green Master Mix (Applied Biosystems), forward and reverse primers (200 nM each), and cDNA derived from 10 ng of total RNA. Plasmids containing each amplicon were used for the calibration curves. The plasmids were constructed with the PCR products and pCR2.1-TOPO vector using a TOPO TA Cloning Kit (Invitrogen, CA, USA). All samples, including cDNA derived from the chicken livers and the plasmid standards, were analyzed in duplicate using the following protocol: 95 °C for 20 s followed by 40 cycles of 95 °C for 3 s and 60 °C for 30 s. At the end of each PCR run, melt curve analysis was performed in the range of 60–95 °C. PCR products were confirmed to be single fragments by electrophoresis and direct sequencing methods. The glyceraldehyde-3-phosphate dehydrongenase (*GAPDH*) gene was used as an internal control. cDNAs obtained without reverse transcription were examined to confirm that the Ct value was >35.

### Enzymatic characterization of chicken CYP2C isoforms using P450-Glo assay

Yeast microsomes expressing chicken *CYP2C23a*, *CYP2C23b*, *CYP2C45* or rat *CYP2C11* were prepared as described previously with several modifications [16]. In brief, the full-length insert encoding chicken *CYP2C23a*, *CYP2C23b*, *CYP2C45* or rat *CYP2C11* was amplified by PCR. The resulting fragments were subcloned into a pGYR1 expression vector containing yeast P450 reductase using In-Fusion HD Cloning Kit (Takara Bio, Japan). The AH22 strain of *Saccharomyces cerevisiae* was transformed with the expression plasmids by the lithium acetate method. The transformants with pGYR/CYP were selected by the complementation of lue2 auxotrophy. The selected transformants were cultivated in synthetic minimal medium (2% (w/v) D-glucose and 0.67% (w/v) yeast nitrogen base without amino acids) supplemented with 20 mg/l histidine. Microsomal fractions of yeast were prepared by the method of Oeda et al. [17]. The P450 content of yeast microsomes was estimated using the method of Omura and Sato [18]. The protein concentration of the microsomes was determined with the bicinchoninic acid (BCA) protein assay reagent (Nacalai Chemical Co., Japan) using bovine serum albumin (BSA) as a standard.

The activity of the cDNA-expressed proteins of chicken CYP2C23a, CYP2C23b, CYP2C45 and rat CYP2C11 were determined by P450-Glo^TM^ CYP2C8 assay and CYP2C9 assay (Promega Corporation, USA) utilizing a demethylation reaction with luciferin 6’ methyl ether (luciferin-ME; 100 µM) and a hydroxylation reaction with 6’ deoxyluciferin (luciferin-H; 100 µM) as the substrates. The assays were performed according to the manufacturer’s instructions with slight modifications. In brief, the test sample contained 1 pmol microsomal CYP, 50 mM phosphate buffer, and 100 µM of either luciferin-ME or luciferin-H. The samples were plated into 96-well white opaque polystyrene plate, pre-incubated at 37 °C for 10min, to which a NADPH regenerating system was added, then incubated for another 30min. After the reaction, 50 µl of reconstituted luciferin reagent was added and incubated at room temperature for further 30 min, and then the relative luminescence intensity was measured. Blank samples were incubated with the addition of phosphate buffer instead of NADPH regenerating system. The experiments were carried out in triplicate.

### Calculation and statistics

Gene copy numbers were calculated for each cDNA sample using standard curves. The copy numbers were normalized with *GAPDH* copy numbers, and the normalized gene expression levels were compared as shown in the figures. The statistical analysis was performed using JMP 9 (SAS Institute, USA). Significant differences were evaluated by Student t test for mRNA induction, and tukey’s HSD test for CYP2C enzymatic activities.

## Results and Discussion

### Avian *CYP1-3*: annotation, nomenclature, and overview of the genes

In this study, we focused principally on the putatively functional genes with full-length and/or intact sequences. Based on phylogenetic and synteny analyses, the avian *CYP1-3* genes were classified as shown in Tables S2–S4. The human *CYP* genes used in the analyses are shown in Table S5. The numbers of genes in each *CYP1-3* family are shown in Table 1. The number of genes in each family did not differ much among the species, while CYP2 family appeared to include many more genes than the CYP1 and CYP3 families. Thomas [19] demonstrated that xenobiotic-metabolizing *CYP* genes have generally gone through rapid birth-death evolution (frequent gene duplications and losses). Our results showed that the avian *CYP2* family genes, same as the human and mouse genes, have undergone frequent duplication events compared with the avian *CYP1* and *CYP3* family genes. However, the complement of *CYP* genes shown here may be incomplete and some genes could not be localized in synteny analysis. Due to the incompleteness of current assembly, annotation and locus information, there’s a possibility that our analysis could not fully cover the CYP complements. The information may be updated in future.

**Table 1:** *CYP1-3* gene numbers in each species.

Family	Chicken	Finch	Turkey	Human
1	4	3	3	3
2	21	16	18	16
3	2	2	2	4
Total	27	21	23	23

### *CYP1*

The *CYP1* phylogeny was constructed with 243 amino acid residues using the JTT matrix-based model (Figure S1). Human *CYP2A13* was used as an outgroup. *CYP1A4/5* and *CYP1B1* genes were confirmed to be completely conserved among the three avian species and human by phylogeny and synteny analyses (Figure S1). *CYP1C1* was found to be a pseudogene with stop codons in zebra finch, turkey, and human CDSs. Only chicken *CYP1C1* was a full-length sequence without stop codons. Although the avian *CYP1A* has been named *CYP1A4/5*, it is orthologous to human *CYP1A1/2* [20].

### *CYP2*

**Figure 1:**
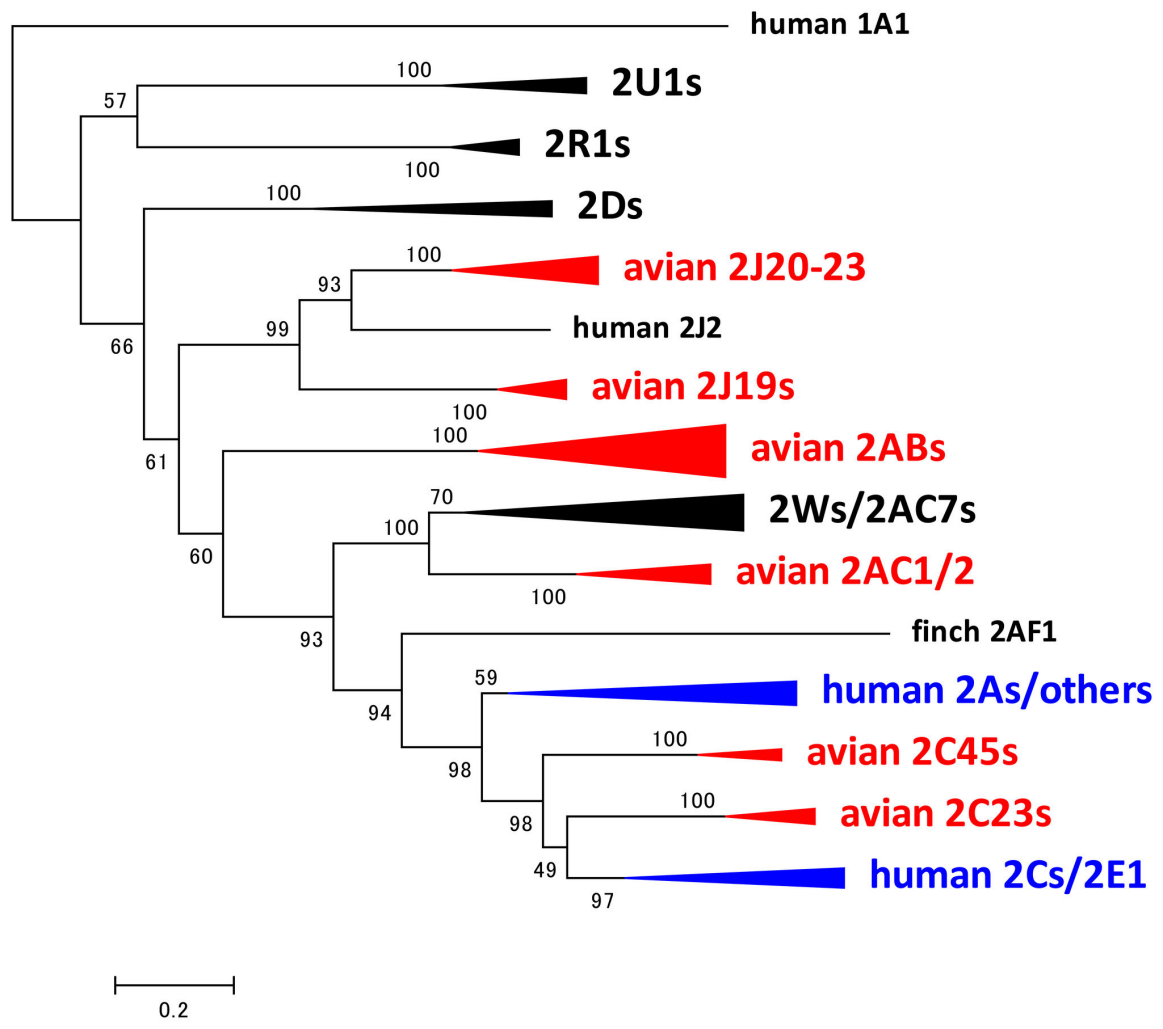
Compressed phylogeny of *CYP2* genes. Phylogeny of CYP2 amino acid sequences from chicken, zebra finch, turkey, and human. The maximum likelihood tree was created using MEGA5 software. The numbers on the branches indicate the number of times per 100 bootstrap replicates that the branch appeared in the trees, estimated by a random resampling of the data. The scale bar represents 20 substitutions per 100 residues. The triangles indicate genes in the same subfamily. The size of the triangles indicates the number of genes included in the branch. Blue triangle and red triangle indicate avian specific branch and human specific branch, respectively. The detailed phylogeny is shown in Figure S2.

The *CYP2* phylogeny was constructed with 441 amino acid residues using the JTT matrix-based model (Figure 1 for a compressed phylogeny, and Figure S2 for a full phylogeny). Human *CYP1A1* was used as an outgroup. The phylogeny was generally consistent with the previously reported *CYP2* phylogeny [9]. Some avian characteristics were found in the *CYP2* family, i.e., multiple genes in the avian *CYP2J*, *CYP2AB*, *CYP2AC*, and *CYP2W* subfamilies, whereas humans have only a single gene, a single pseudogene in these subfamilies, absence of *CYP2B* and *CYP2E*; and a unique duplication pattern of *CYP2C* genes as described below.

### *CYP2D*, *CYP2R*, and *CYP2U*

**Figure 2:**
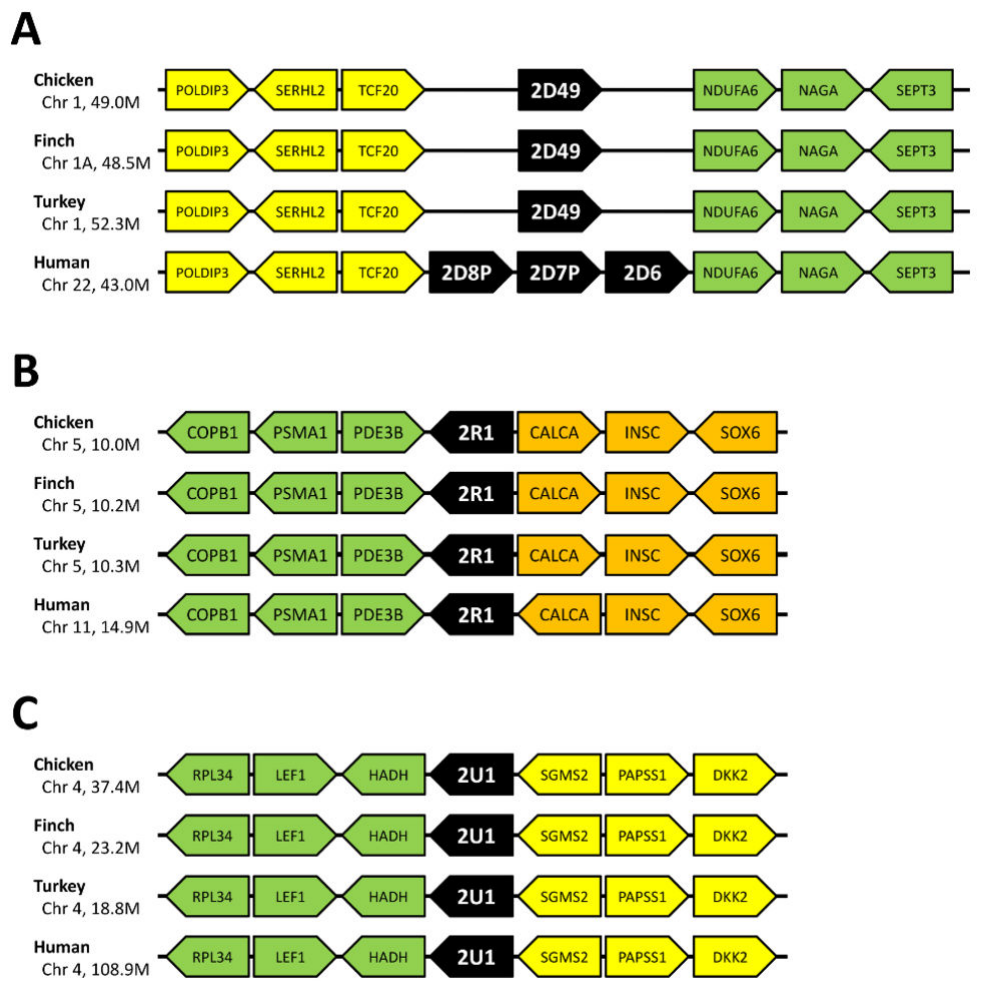
Typical fully shared synteny of *CYP2D, 2R* and *CYP2U*. Synteny was well conserved for the following genes (A) *CYP2D* *genes*. (B) *CYP2R1*, (C) *CYP2U1*. CYP2R1 and CYP2U1 are known to metabolize endogenous compounds in mammal, while chicken CYP2D49 has been reported to metabolize bufuralol, a beta blocker drug.

There were single genes in the *CYP2D*, *CYP2R*, and *CYP2U* subfamilies in all species. Synteny was fully shared for *CYP2R1* and *CYP2U1* among birds and humans (Figure 2). The *CYP2D* cluster was also conserved among the species, except that the human *CYP2D* cluster contains *CYP2D6* and two other *CYP2D* pseudogenes between *TCF20* and *NDUFA6*. Because CYP2R and CYP2U have essential functions in the metabolism of the endogenous substrates vitamin D and arachidonic acid, these genes may be strongly conserved [21], [22](Chuang, 2004 #66).

### *CYP2ABFGST* cluster

In human and mouse, the *CYP2ABFGST* clusters are known to be located on chromosomes 19 and 7A3, respectively [15]. Kirischian et al. [23] previously suggested that CYP2A, CYP2B, CYP2F, CYP2G, and CYP2S were mammalian-specific. In our investigation, neither the *CYP2ABFGST* cluster nor any of the genes included in the cluster, such as *CYP2A* and *CYP2B*, were found with the search tools in any of the avian species (data not shown). The anole lizard is known to possess the *CYP2G* gene. Thus, the *CYP2ABFGST* cluster may have been lost only in the avian lineage.

### CYP2C/2E

**Figure 3:**
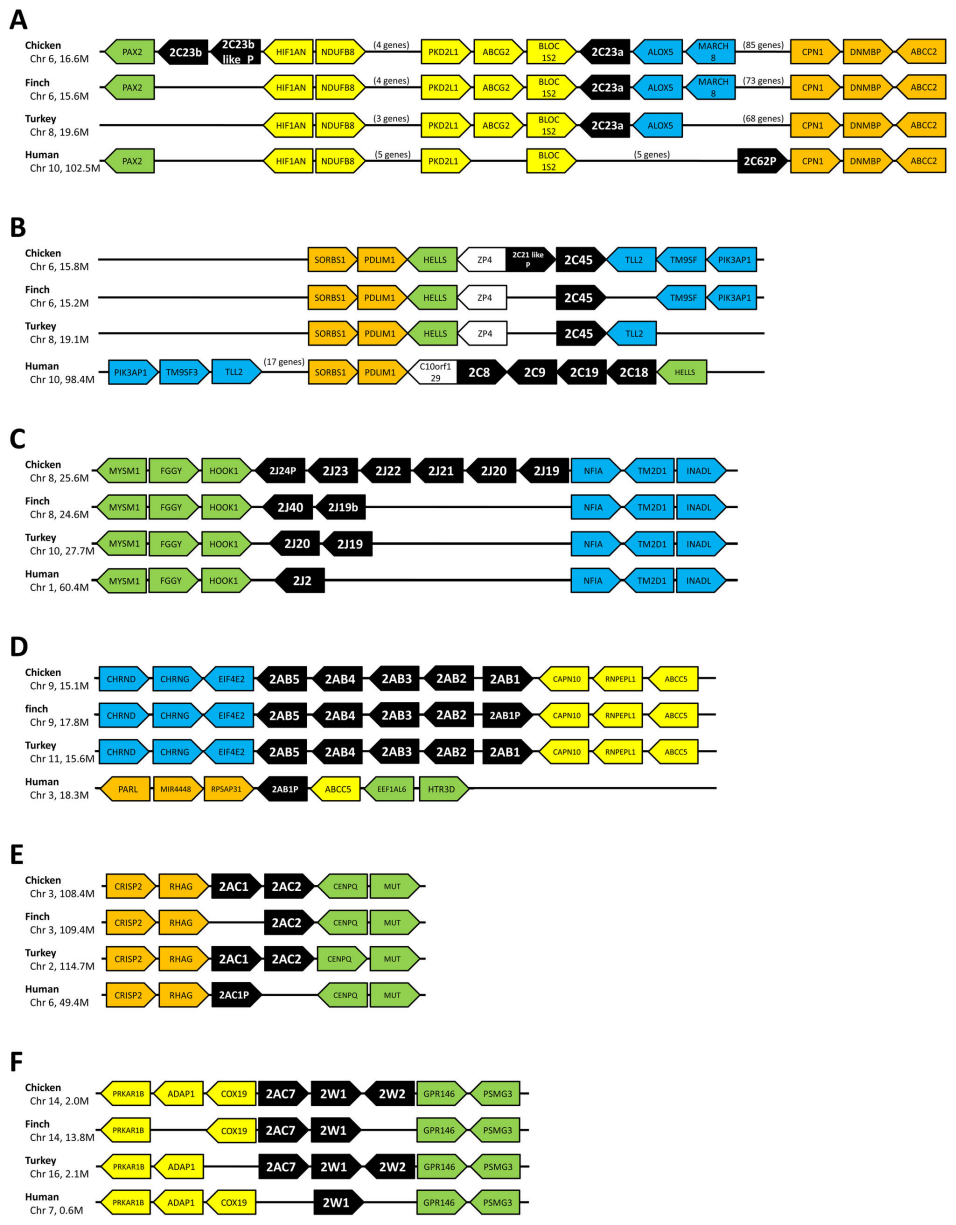
Synteny of *CYP2* family genes. (A) Synteny of *CYP2C23* genes. Only chicken had two *CYP2C23* genes, whereas the other two birds possessed only one *CYP2C23a* gene. Avian *CYP2C23* genes have been reported to be orthologous to human *CYP2C62P*, rat *CYP2C23* and mouse *CYP2C44*. (B) Synteny of *CYP2C45* genes. *CYP2C45* genes were generally conserved among bird species. Although both avian *CYP2C45* genes and human *CYP2C* genes located nearby SORBS1-PDLIM1 cluster, phylogeny did not suggest that avian *CYP2C45* genes are related to human CYP2C genes. These results implied that the avian and human CYP2C genes having gone through independent duplication events in each lineage. (C) Synteny of *CYP2J* genes. Two of the zebra finch *CYP2J* genes were not localized to the chromosome and are not shown in this figure. (D) Synteny of *CYP2AB* genes. Five avian CYP2AB genes were fully conserved among bird species, while human have only one pseudogene. (E) Synteny of *CYP2AC1* and *CYP2AC2* genes. While avian species possessed one or two intact *CYP2AC* genes, human possessed only one pseudogene. The synteny was generally shared among bird species and human. (F) Synteny of *CYP2AC7* and *CYP2W* genes. The gene orders around *CYP2AC7* and *CYP2W* genes were generally conserved among species, although bird species possessed another *CYP2W* gene and *CYP2AC7* gene in addition to *CYP2W1*. *CYP2AC7* genes were thought to have diverged from CYP2W genes, and became a parent gene for CYP2AC1 and CYP2AC2.

Avian *CYP2C* genes were formerly called *CYP2H* genes because they were assumed to be avian-specific. However, Kubota et al. [9] reported that the genes corresponded to mammalian *CYP2C* genes by phylogeny and synteny analyses. In our phylogeny, the avian *CYP2C* subfamily also comprised a clade with the human *CYP2C* and *CYP2E* clade. Human *CYP2Cs* and *CYP2E1* together formed a single clade, which is clearly distinct from the avian *CYP2C23* and *CYP2C45* clade. The synteny of *CYP2C23* and *CYP2C45* are shown separately in Figure 3, although their positions are <1 Mb apart. In the *CYP2C23* locus, *CYP2C23a* was located between *BLOC1S2* and *ALOX5* in the three bird species, whereas only chicken possessed another gene, *CYP2C23b* and *CYP2C23b*-like pseudogene between *PAX2* and *HIF1AN*. The positional relations of *PAX2*, *HIF1AN*, *BLOC1S1*, and *CPN1* were also conserved in human, but the *CYP2C62* pseudogene was located next to *CPN1*. In turkey, a *CYP2C23*-like partial sequence existed between *ALOX5* and *CPN1*. This sequence is assumed to have stop codons in CDS. In birds, >70 genes are present between *BLOC1S2* and *CPN1* genes; however, in human, only six genes including *CYP2C62p* are present between the *BLOC1S2* and *CPN1* genes.

The synteny of *CYP2C45* was the most disrupted among the genes examined in this study. In chicken, *CYP2C45* with a *CYP2C21*-like pseudogene was located 0.8 Mb upstream of the *CYP2C23* locus. The avian *CYP2C45* genes were located between *SORBS1-PDLIM-HELLS-ZP4* and *TLL2-TM9SF3*. In contrast, *CYP2C*s, i.e., *CYP2C8*, *CYP2C9*, *CYP2C18*, and *CYP2C19*, were located between *SORBS1-C10orf129* and *HELLS* in human, and the *TM9SF3-TLL2* cluster was located on the opposite side compared with the avian species. The human *CYP2E1* gene was located between *OR7M1P* and *SYCE1* at 135.3 Mb on chromosome 10, whereas *CYP2C* genes were located at 96.4–101.9 Mb on the same chromosome. The corresponding region and clusters to human CYP2E1 locus were not found in avian species.

From the above analyses, *CYP2C* genes and the cluster did not share clear synteny between avian species and human, whereas the synteny was shared among the avian species. Thus, clear orthologous relationships are not suggested between the avian and human *CYP2C* genes. This was also supported by the phylogeny. Rather, it is suggested that a single *CYP2C* ancestor gene duplicated independently in the avian and human lineages to form the current *CYP2C* gene complements. The three *CYP2C23* genes, including one pseudogene only in chicken, suggest that duplication of the *CYP2C23* gene may have occurred once or twice in early chicken history. This implies that the avian *CYP2C* genes loci have undergone frequent alteration in the genome structure.

### *CYP2J*

Humans have only one *CYP2J* gene, whereas avian species have multiple *CYP2J* genes. We searched for the *CYP2J* genes not only in GenBank but also in Ensembl and UCSC BLAT. We found that chicken has six genes including one partial sequence. Zebra finch has four genes, although two of them are not localized on the chromosome. Turkey has two genes and one partial gene. Synteny analysis showed that *CYP2J* genes were conserved on the chromosome, i.e., between *MYSM1-FGGY-HOOK1* and *NFIA-TM2D1-INADL* (Figure 3). The phylogeny suggested independent duplication events in the early chicken and zebra finch lineages.

### *CYP2AB*

The *CYP2AB* clade was constructed with only bird genes because humans have only a *CYP2AB1* pseudogene, and it was excluded from phylogenetic analysis. The *CYP2AB* genes were divided into two clades, the *CYP2AB1*, *CYP2AB4*, and *CYP2AB5* clade and the *CYP2AB2* and *CYP2AB3* clade. *CYP2AB1* through *CYP2AB5* genes were found in all avian species, although zebra finch *CYP2AB1* was found to be a pseudogene with a stop codon in CDS. Turkey *CYP2AB3* was not found in GenBank but was found in Ensembl.

In synteny analysis, avian *CYP2AB* genes were found to be fully conserved between *CHRND-CHRNG-EIF4E2* and *CAPN10-RNPEPL1-ABCC5*, although the human *CYP2AB1* pseudogene was found between *PARL-MIR4448-RPSAP31* and *ABCC5-EEF1AL6-HTR3D* on chromosome 3 (Figure 3). In human, the *CHRND-CHRNG-EIF4E2* cluster and *CAPN10-RNPEPL1* cluster were found at 233.4 Mb and 241.5 Mb, respectively, on chromosome 2.

### *CYP2AC1/2*

*CYP2AC1* and *CYP2AC2* formed a clade with a sister clade of *CYP2W* and *CYP2AC7* in the phylogeny. *CYP2AC1* and *2AC2* genes were found between *RHAG* and *CENPQ* in each species, and the synteny was clearly conserved including in human (Figure 3). In human, only a single *CYP2AC1p* was found in the corresponding locus. Zebra finch possessed only one *CYP2AC2* gene, and chicken and turkey possessed two *CYP2AC* genes, *CYP2AC1* and *CYP2AC2*. This situation with fully conserved synteny and few duplication events is similar to that of *CYP2D*, *CYP2R*, and *CYP2U*, although functions or physiological roles have not yet been reported for *CYP2AC1* and *CYP2AC2*.

### *CYP2AC7/2W1*

*CYP2AC7* and *CYP2W* genes formed one clade in the phylogeny with a sister clade of *CYP2AC1* and *CYP2AC2* genes.

In synteny analysis, the *CYP2AC7* and *CYP2W* genes were located between *PRKAR1B-ADAP1-COX19* and *GPR146-PSMG3* in birds and humans (Figure 3). In chicken and turkey, there were two *CYP2W* genes (*CYP2W1* and *CYP2W2*) and one *CYP2AC7* gene, whereas in zebra finch, there was only one *CYP2W* gene. Only one *CYP2W1* gene was present in human. Duplication events only in the Galloanserae lineage are suggested for the *CYP2W1* and *CYP2W2* genes. *CYP2AC7* may be a parent gene for *CYP2AC1/2* genes.

### *CYP3*

**Figure 4:**
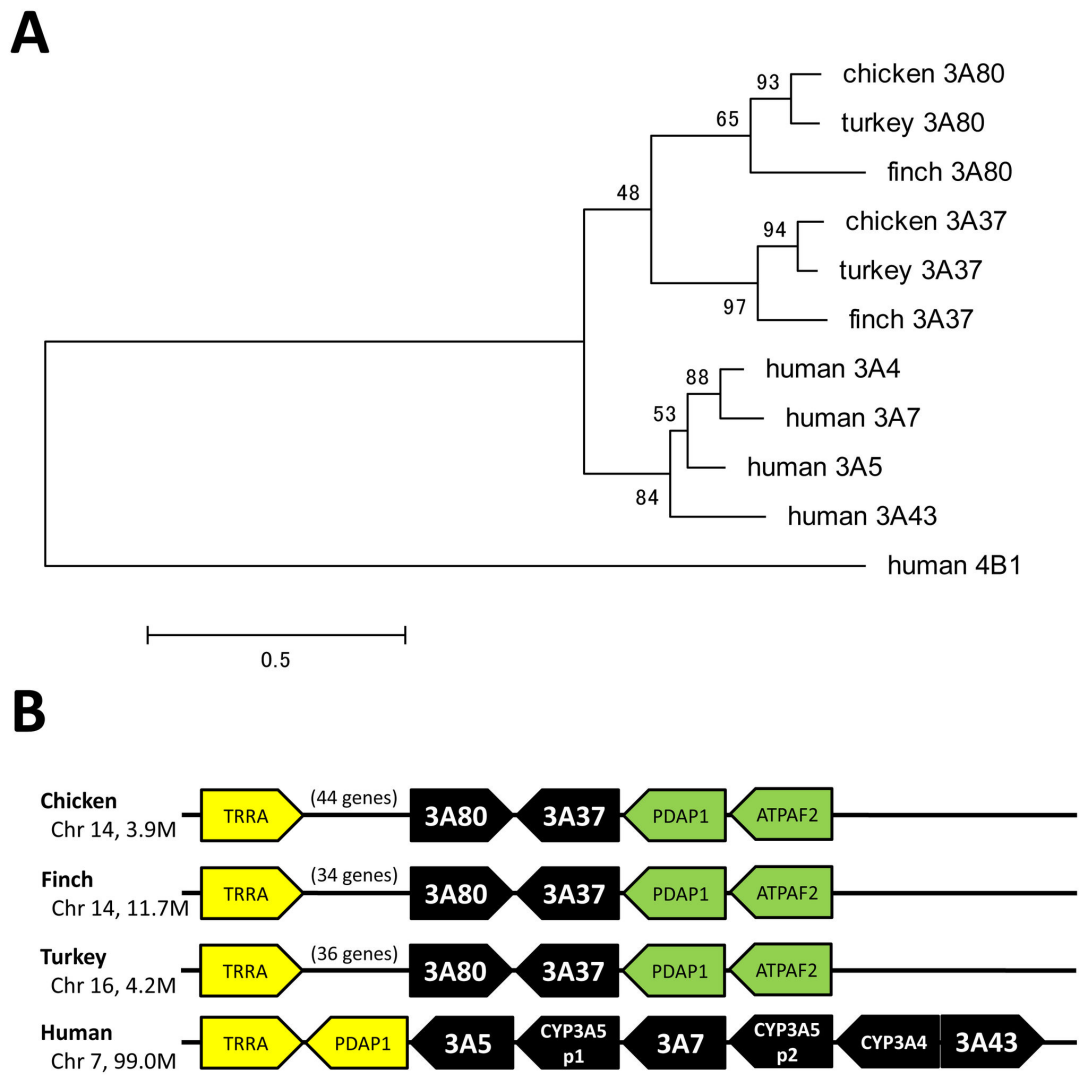
Phylogenetic tree and synteny of CYP3A genes. (A) Phylogeny of CYP3 amino acid sequences from chicken, zebra finch, turkey, and human. The maximum likelihood tree was created using MEGA5 software. The numbers on the branches indicate the number of times per 100 bootstrap replicates that the branch appeared in the trees, estimated by a random resampling of the data. The scale bar represents 50 substitutions per 100 residues. (B) Avian species possessed two *CYP3A* genes, whereas humans possessed six genes including two pseudogenes. Although the loci of *CYP3A* genes were almost conserved between avian species and human, none of the avian *CYP3A* genes showed clear correspondence to any of the human *CYP3A* genes.

The phylogeny was constructed with 315 amino acid residues using the JTT matrix-based model (Figure 4). Human *CYP4B1* was used as an outgroup. The *CYP3* family was clearly divided into two clades, the bird *CYP3A* clade and human *CYP3A* clade, as shown in the phylogeny. Two *CYP3A* genes, *CYP3A37* and *CYP3A80*, are present in birds, whereas four *CYP3A* genes are present in humans.

In birds, the gene order around *CYP3A37* and *CYP3A80* was fully conserved among species because there were more than 34–44 genes between *TRRA* and *CYP3A80-CYP3A37-PDAP1* (Figure 4). In contrast, in human, *PDAP1* was located next to *TRRA* and the *CYP3A* genes were arranged next to them in tandem. This suggests that a chromosome inversion occurred around the locus of the *CYP3A* gene in either the avian or human lineage. The phylogenetic and synteny analyses suggested no clear orthology between any of the bird *CYP3A* genes and any of the human *CYP3A* genes. McArthur et al. [24] suggested independent *CYP3A* duplication events in early rodent history, primate history, and teleost history. Our results also suggested a single duplication event of the *CYP3A* gene in early bird history.

### mRNA expression levels in chicken liver; basal expression and induction by PB

**Figure 5:**
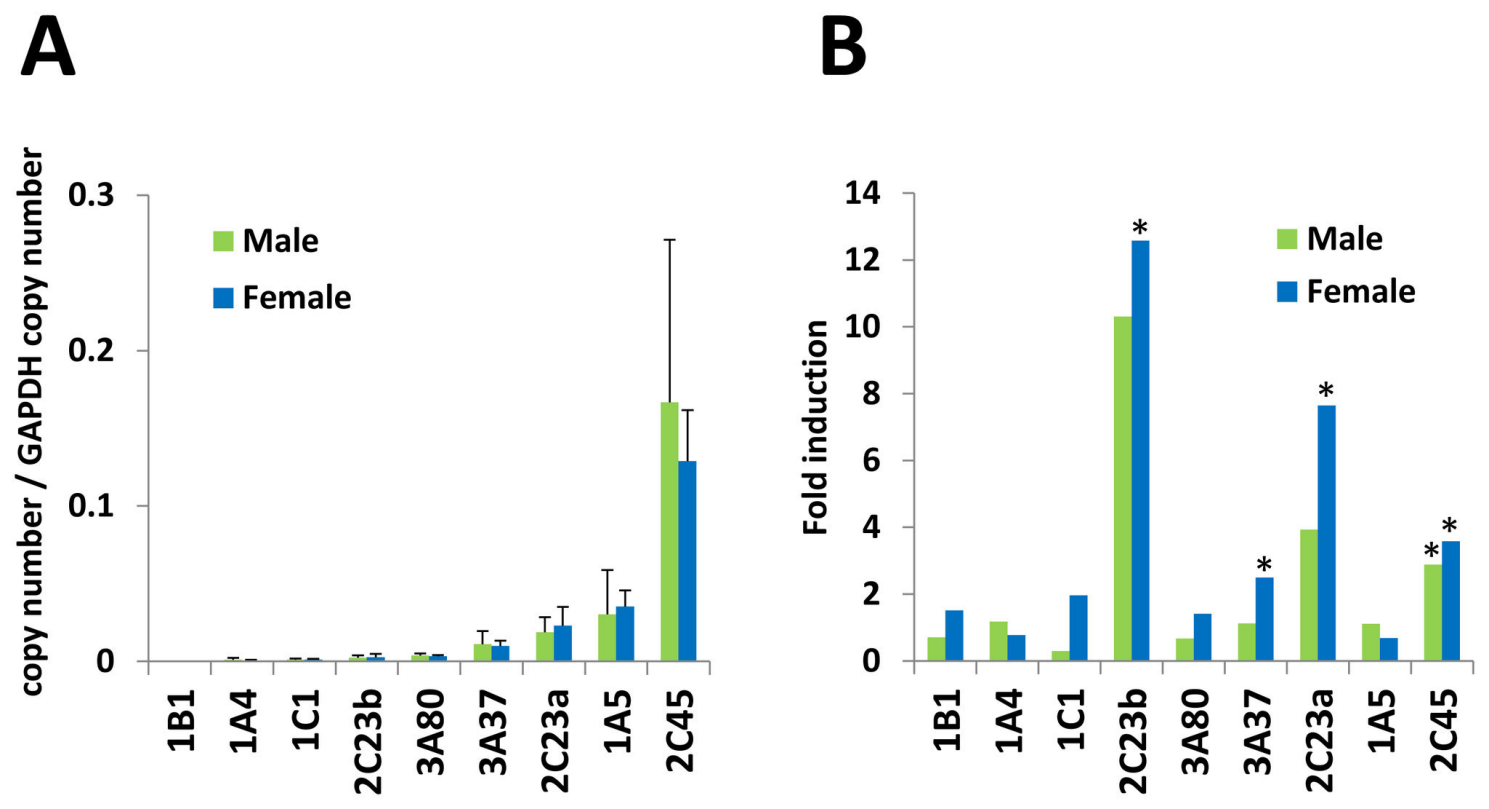
Comparison of basal mRNA expression levels and induction by PB of *CYP* genes in chicken liver. qRT-PCR was performed with cDNAs derived from chicken liver for following genes: *CYP1A4*, *CYP1A5*, *CYP1B1*, *CYP1C1*, *CYP2C23a*, *CYP2C23b*, *CYP2C45*, *CYP2D49*, *CYP3A37*, and *CYP3A80*. The transcripts of each gene were calculated using plasmid standards. The values represent the expression levels normalized with *GAPDH* gene transcripts. (A) basal expression levels in non-treated chicken liver. Data are expressed as an average ± SD (B) fold induction levels by PB are indicated by the ratio of the mRNA expression levels of PB-treated chicken liver and saline-treated chicken liver for each gene. The fold induction level is shown by an average, and the significant induction of genes in PB-treated chicken compared to saline-treated chicken is indicated by an asterisk. N=4 for each group.

We focused on the isoforms that have been reported in the literature or are putatively predominant in liver. Ten isoforms, *CYP1A4*, *CYP1A5*, *CYP1B1*, *CYP1C1*, *CYP2C23a*, *CYP2C23b*, *CYP2C45*, *CYP2D49*, *CYP3A37*, and *CYP3A80*, were examined. We examined *GAPDH* and *beta-actin* for normalization and found that *GAPDH* was more stable, including in phenobarbital-induced chicken liver samples (data not shown). In the above gene set, *CYP2C45* showed the highest expression level in both male and female chickens, followed by *CYP1A5*, *CYP2C23a*, and *CYP2D49* (Figure 5). The *CYP3A* isoforms, i.e., *CYP3A37* and *CYP3A80*, had the fifth and sixth highest expression levels in this gene set. In chicken, *CYP2C45*, which showed the highest mRNA expression, is suggested as a candidate for the most important isoform. In contrast, chicken *CYP3A37* and *CYP3A80* showed relatively low expression. Blevins et al. [25] have reported the age-related alteration in 7-benzyloxyquinoline-metabolizing activity, which is considered to be a function of CYP3A proteins. The enzymatic activity was highest in 8-week-old chickens, and at 20 weeks, the activity had declined to one-fifth of the activity at 8 weeks. Because our samples in this study were aged 8 weeks, older or adult chickens may have even lower mRNA expression levels of *CYP3A* genes. *CYP3A37* genes have been cloned and characterized in chicken and turkey, while *CYP3A80* have not been characterized in any literature, to our knowledge [11,26]. This is consistent with our result, which suggested *CYP3A80* has far lower expression levels in chicken liver. Thus, *CYP3A37* was suggested to be more important in avian xenobiotic metabolism than *CYP3A80* because of the higher expression level. In our qRT-PCR, none of the genes examined showed sex differences in mRNA expression levels. However, age-related alterations may possibly affect mRNA expression patterns in chicken liver. This study is the first to report mRNA expression profiles of *CYP1-3* genes in chicken liver. Further study is needed to elucidate the relationships among mRNA expression levels, protein expression levels, and enzymatic activity and their alterations by factors such as inducing agents, age and sex. We further investigated the mRNA induction by PB, a typical mammalian CAR activator. However, chicken do not possess CAR or PXR, but chicken xenobiotic receptor (CXR) as an orthologous receptor to them [27]. In chicken, PB is known to induce *CYP2C* genes and *CYP3A* genes [10,11,28,29]. Our result also showed significant induction of *CYP2C23a*, *CYP2C23b*, *CYP2C45* and *CYP3A37* in female chicken, although only *CYP2C45* was significantly induced in male chicken. The rank order of the average induction levels were *CYP2C23b* > *CYP2C23a* > *CYP2C45* > *CYP3A37* in both male and female chicken.

### Function and possible role of CYP2C isoforms in avian xenobiotic metabolism

As the results of mRNA expression levels suggested *CYP2C* genes may have important roles in chicken xenobiotic metabolism, we examined the function of the CYP2C isoforms using P450-Glo assay kit, with substrates of luciferin-H (CYP2C8 assay) and luciferin-ME (CYP2C9 assay). In comparison to rat CYP2C11 as a positive control, all the chicken CYP2C isoforms showed lower activity with luciferin-H, and higher activity in luciferin-ME (Table 2). Among the chicken CYP2C isoforms, the activity were significantly different to both substrates, suggesting the clear difference in substrate selectivity even between CYP2C23a and CYP2C23b which shared 93.4% of identity percentage of amino acid sequences. The rank order of the activity toward luciferin-H and luciferin-ME were both CYP2C23a > CYP2C23b > CYP2C45. Although the activities toward the P450-Glo substrates were lower than the other CYP2C isoforms, CYP2C45 may have larger contribution to xenobiotic metabolism *in vivo* because of the constitutive high expression of mRNA. On the other hand, CYP2C23a and CYP2C23b showed higher induction compared to CYP2C45 by phenobarbital and higher activity toward luciferin-H and luciferin-ME. They may be isoforms important under the exposure of xenobiotic chemicals.

**Table 2:** Activity of CYP2C proteins assayed with P450-Glo.

		**Luciferin-H**				**Luciferin-ME**		
**Chicken CYP2C23a**	196.3	±	6.3	^1^	1869.0	±	63.5	^a^
**Chicken CYP2C23b**	62.7	±	3.8	^b^	236.3	±	9.2	^b^
**Chicken CYP2C45**	12.7	±	3.8	^c^	142.7	±	6.7	^b^
**Rat CYP2C11**	262.7	±	7.9	^d^	121.7	±	6.8	^b^

Activity of CYP2C23a, CYP2C23b and CYP2C45 proteins were examined with P450-Glo assay. The assays were performed in triplicate. Data are expressed as an average ± SE, with a unit of relative luminescent unit. Different characters indicate significant differences.

## Conclusions

In this study, we have established a comprehensive understanding of the existence and overall relationships of avian *CYP1-3* genes in chicken, zebra finch, and turkey in comparison with human genes. We have revealed several remarkable characteristics of avian *CYP1-3* genes, including the absence of clear orthology between avian and human *CYP2C* or *CYP3A* genes and the existence of *CYP2J*, *CYP2AB*, and *CYP2AC* duplication events in the early bird lineage. A common feature among birds and humans was also found, in that *CYP2R1* and *CYP2U1* were fully conserved despite independent avian and mammalian evolution, which is probably because of their essential functions in the metabolism of endogenous compounds. Because species differences in the avian and mammalian *CYP1-3* genes may be important for their extrapolation to the fields of pharmacology and toxicology, detailed enzymatic and physiological characterization of the avian *CYP1-3* genes is required.

In qRT-PCR assays, *CYP2C45* showed the highest basal mRNA expression in chicken liver, and *CYP2C23b* gene was the most induced gene by PB followed by *CYP2C23a* and *CYP2C45*. In the enzymatic characterization of CYP2C isoforms utilizing P450-Glo substrates, the highest activity was observed in CYP2C23a and the lowest in CYP2C45. Because the contribution of each CYP isoform to the drug metabolism is greatly decided by its protein expression levels in liver, avian *CYP2C45* may also be the dominant isoform in avian xenobiotic metabolism, and other CYP2C genes may have a role to respond to intake of xenobiotics. Based on these findings, further investigation is needed to characterize in detail the avian *CYP* isoforms, including their expression patterns in other organs, proteomic approaches to quantifying the proteins, functional analyses, and their relationships with chemical sensitivities.

## Supporting Information

Figure S1 **Phylogenetic tree and synteny of *CYP1* family genes.** (A) Phylogeny of *CYP1* amino acid sequences from chicken, zebra finch, turkey, and human. The maximum likelihood tree was created using MEGA5 software. The numbers on the branches indicate the number of times per 100 bootstrap replicates that the branch appeared in the trees, estimated by a random resampling of the data. The scale bar represents 20 substitutions per 100 residues. (B) *CYP1A4* and *CYP1A5* genes (orthologues of human *CYP1A1* and *CYP1A2*). (C) *CYP1B1* genes.
(TIF)

Figure S2 **Full phylogenetic tree of *CYP2* family genes.** Phylogeny of *CYP2* amino acid sequences from chicken, zebra finch, turkey, and human. The maximum likelihood tree was created using MEGA5 software. The numbers on the branches indicate the number of times per 100 bootstrap replicates that the branch appeared in the trees, estimated by a random resampling of the data. The scale bar represents 50 substitutions per 100 residues. The compressed version of this phylogeny is shown in Figure 1.
(TIF)

Table S1 **Primers for qRT-PCR.**
(XLS)

Table S2 **CYP 1-3 genes of chicken.**
(XLSX)

Table S3 **CYP 1-3 genes of zebra finch.**
(XLSX)

Table S4 **CYP 1-3 genes of turkey.**
(XLSX)

Table S5 **CYP 1-3 genes of human.**
(XLSX)
