# Supplementary figures and images for: Avian Cytochrome P450 (CYP) 1-3 Family Genes: Isoforms, Evolutionary Relationships, and mRNA Expression in Chicken Liver
Source: PLoS One. 2013 Sep 30;8(9):e75689. doi: 10.1371/journal.pone.0075689 (PMC3786927; doi:10.1371/journal.pone.0075689)

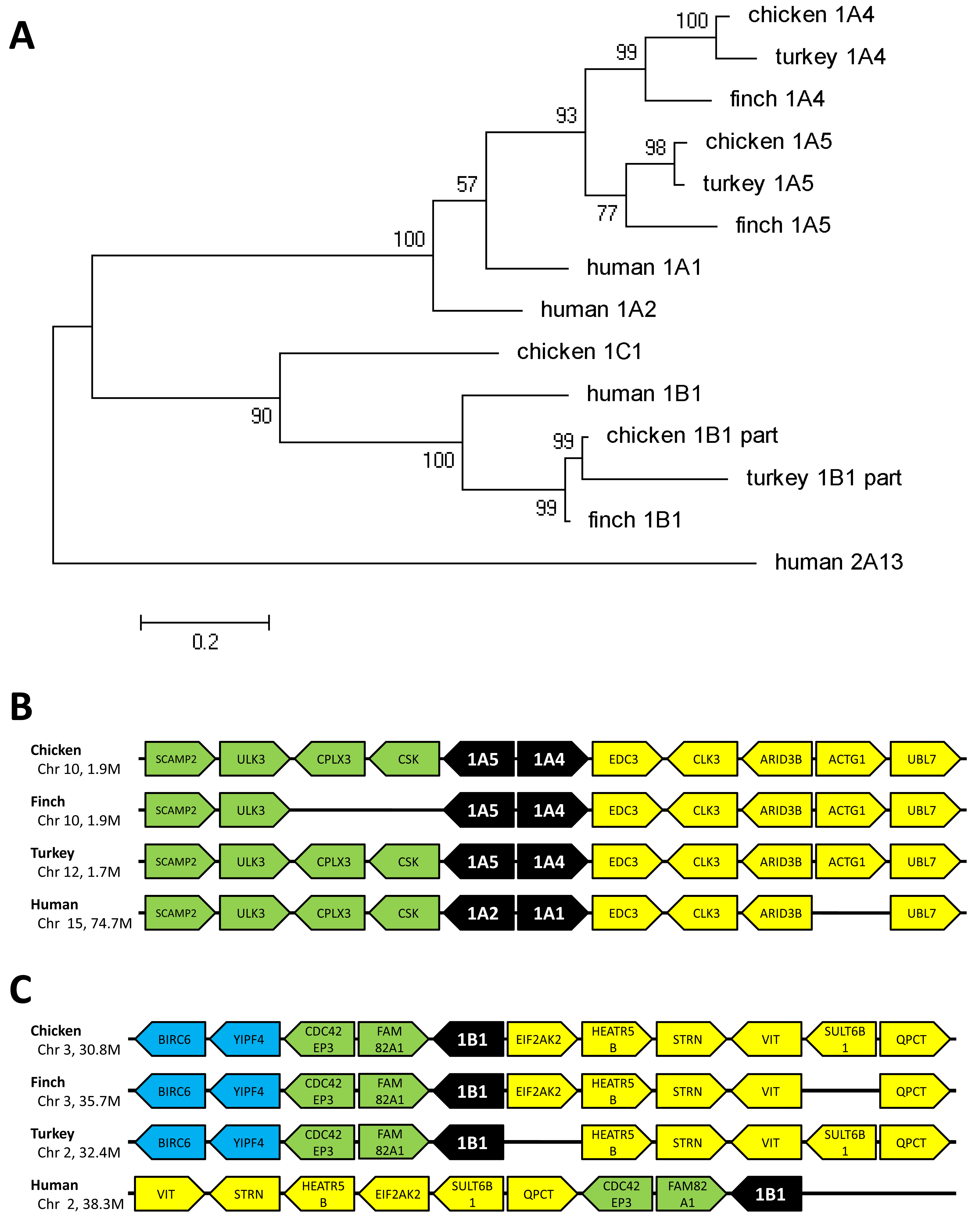

Supplement: Figure S1 — Phylogenetic tree and synteny of CYP1 family genes. (A) Phylogeny of CYP1 amino acid sequences from chicken, zebra finch, turkey, and human. The maximum likelihood tree was created using MEGA5 software. The numbers on the branches indicate the number of times per 100 bootstrap replicates that the branch appeared in the trees, estimated by a random resampling of the data. The scale bar represents 20 substitutions per 100 residues. (B) CYP1A4 and CYP1A5 genes (orthologues of human CYP1A1 and CYP1A2). (C) CYP1B1 genes. (TIF) [file pone.0075689.s001.tif]

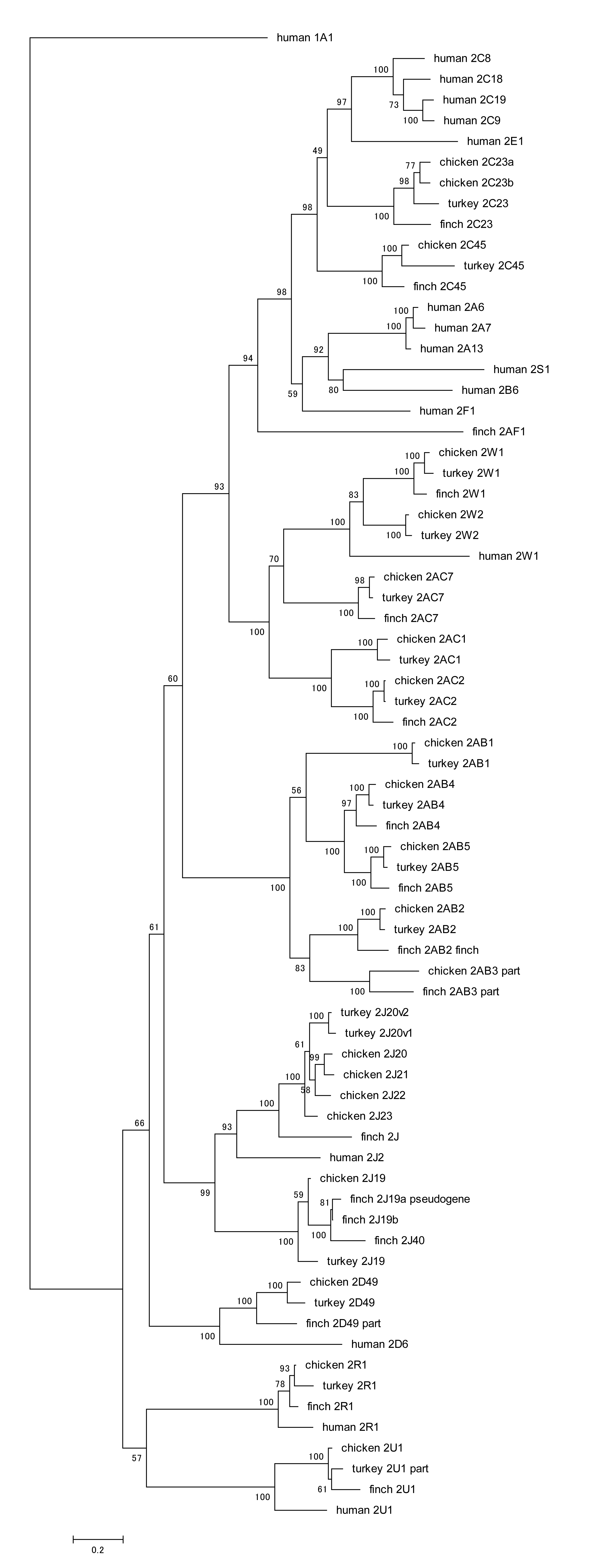

Supplement: Figure S2 — Full phylogenetic tree of CYP2 family genes. Phylogeny of CYP2 amino acid sequences from chicken, zebra finch, turkey, and human. The maximum likelihood tree was created using MEGA5 software. The numbers on the branches indicate the number of times per 100 bootstrap replicates that the branch appeared in the trees, estimated by a random resampling of the data. The scale bar represents 50 substitutions per 100 residues. The compressed version of this phylogeny is shown in Figure 1. (TIF) [file pone.0075689.s002.tif]
